# Locomotion Controls Spatial Integration in Mouse Visual Cortex

**DOI:** 10.1016/j.cub.2013.04.012

**Published:** 2013-05-20

**Authors:** Aslı Ayaz, Aman B. Saleem, Marieke L. Schölvinck, Matteo Carandini

**Affiliations:** 1 UCL Institute of Ophthalmology, University College London, London EC1V 9EL, UK

## Abstract

Growing evidence indicates that responses in sensory cortex are modulated by factors beyond direct sensory stimulation [1–8]. In primary visual cortex (V1), for instance, responses increase with locomotion [9, 10]. Here we show that this increase is accompanied by a profound change in spatial integration. We recorded from V1 neurons in head-fixed mice placed on a spherical treadmill. We characterized spatial integration and found that the responses of most neurons were suppressed by large stimuli. As in primates [11, 12], this surround suppression increased with stimulus contrast. These effects were captured by a divisive normalization model [13, 14], where the numerator originates from a central region driving the neuron and the denominator originates from a larger suppressive field. We then studied the effects of locomotion and found that it markedly reduced surround suppression, allowing V1 neurons to integrate over larger regions of visual space. Locomotion had two main effects: it increased spontaneous activity, and it weakened the suppressive signals mediating normalization, relative to the driving signals. We conclude that a fundamental aspect of visual processing, spatial integration, is controlled by an apparently unrelated factor, locomotion. This control might operate through the mechanisms that are in place to deliver surround suppression.

## Highlights

• Spatial integration in neurons of mouse visual cortex depends on locomotion
• Locomotion increases the region of visual space that V1 neurons integrate
• Locomotion reduces surround suppression
• Locomotion could achieve this by adjusting the strength of divisive normalization

## Graphical Abstract

**Figure:**
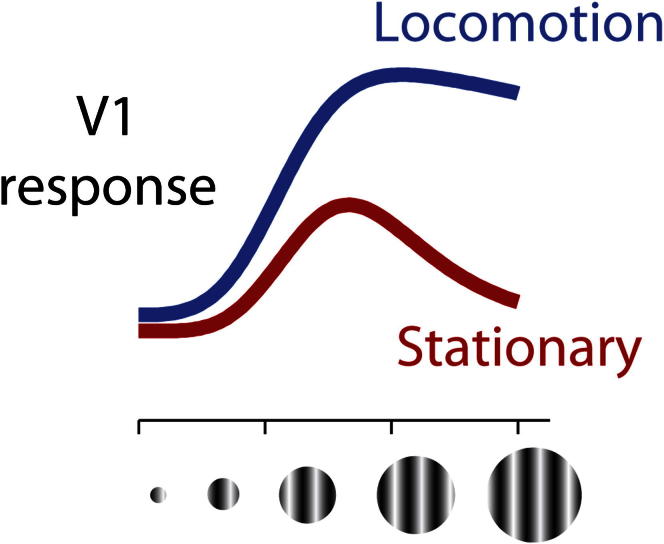

## Results and Discussion

**Figure 1:**
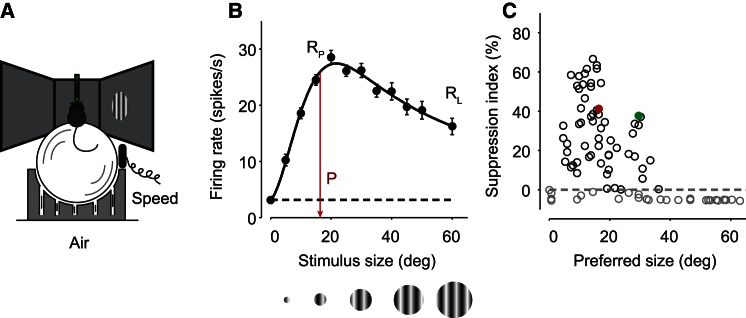
Size Tuning in Mouse V1 (A) Head-fixed mice were placed on an air-suspended spherical treadmill. Extracellular activity was recorded using multilaminar silicon probes while drifting gratings were presented on the screen. (B) Size tuning response of an example neuron. Curve shows the model fit, and error bars are the SEM. P indicates preferred stimulus size, where response reaches 95% of peak response. R_P_ and R_L_ indicate responses at preferred and largest stimulus sizes. (C) Population suppression indices for all recorded neurons (n = 89). Black dots indicate size-tuned neurons, which have positive suppression indices. Red dot is the neuron in (B); green dot indicates the neuron in Figure 2A.

We started by characterizing the properties of spatial integration and found that most neurons in area V1 of awake mice showed clear surround suppression (Figure 1). We recorded multiunit activity and single-unit activity from V1 while mice were head-restrained on an air-suspended ball [9] (Figure 1A). The visual stimuli were gratings centered on the receptive fields of the recorded neurons, varying randomly in size. Consistent with previous reports in monkey [15] and mouse [16–18], the responses of most neurons showed first an increase with increasing size, followed by suppression as the grating size exceeded an optimal value (e.g., Figure 1B). To quantify the strength of this surround suppression, we defined a suppression index as (R_p_ – R_L_)/R_L_, where R_L_ and R_P_ are the responses to the largest size and to the preferred size. For robustness, we defined the latter as the smallest size that elicited >95% of the maximal response (Figure 1B). Based on suppression index and preferred stimulus size, the neurons in the population could be roughly divided into two groups (Figure 1C). The majority (60 of 89) showed preferred sizes in the range of 16° ± 5° (median ± median absolute deviation) and positive suppression indices (30% ± 13%, Figure 1C, black dots). The rest (29 of 89) showed no evident suppression: their suppression indices were negative, indicating that the size tuning curves saturated or kept on growing with increasing size (Figure 1C, gray dots). These cells might have showed surround suppression if we had been able to show larger stimuli. Indeed, the display spanned only 45° vertically, so our largest stimuli (60° diameter) were partially clipped and larger stimuli were not feasible. In the following, we report results for both groups, but our interest is in the cells where we could measure surround suppression.

**Figure 2:**
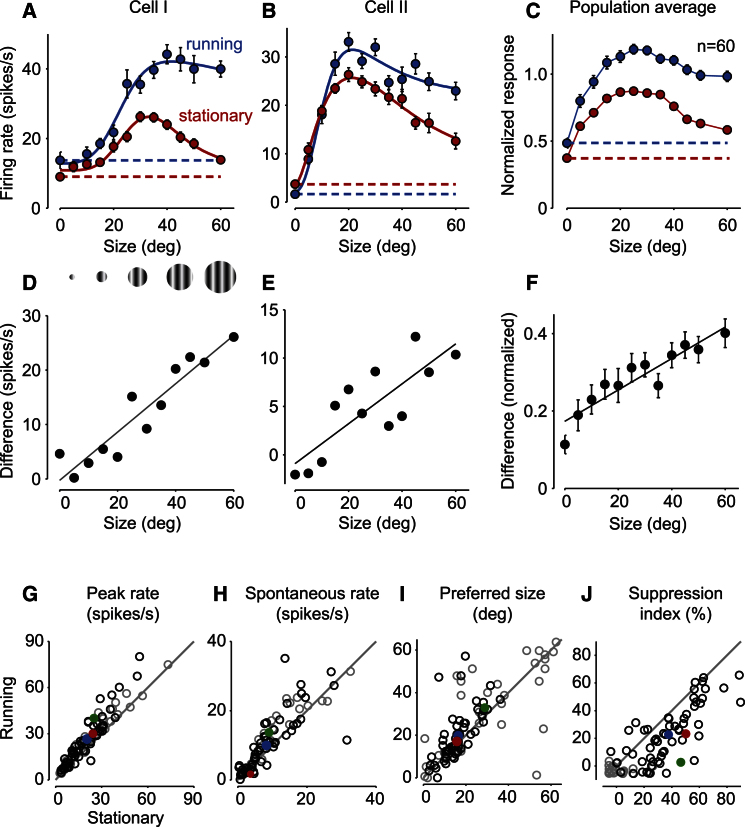
Locomotion Reduces Surround Suppression (A and B) Response of two example neurons while the mouse was stationary (red) and during locomotion (blue). Dotted lines show the spontaneous firing rate. Error bars represent SEM. (C) The average responses of size-tuned neurons (n = 60), normalized to the peak stationary response. Red and blue indicate stationary and running. Error bars represent SEM. (D) The effect of locomotion grows with stimulus size. Ordinate/vertical axis indicates the difference between responses during locomotion and when stationary, for the example cell in (A). Diagonal line indicates linear fit. (E and F) Differences between responses at locomotive and stationary states for the example cell in (B) and for the average responses in (C) (R^2^ = 0.86). Error bars represent SEM. (G) Effect of locomotion on peak firing rate for all recorded neurons. Open black circles represent size-tuned neurons (n = 60); blue circle indicates the mean of these values. Gray circles represent neurons that were not size tuned (suppression index < 0, n = 29). Green and red dots indicate the example neurons in (A) and (B). (H–J) Locomotion effects on spontaneous firing rate (H), preferred stimulus size (I), and suppression index (J). See also Figures S1–S4.

Locomotion strongly decreased the strength of surround suppression (Figure 2). Animals typically spent ∼51% ± 22% (SD, n = 4 mice, 9 sessions) of the time being stationary (speed ≤ 1.2 cm/s). During these periods, cells typically showed strong surround suppression (e.g., Figure 2A, red, suppression index = 47%). During locomotion, however, this surround suppression was typically greatly reduced (e.g., Figure 2A, blue, suppression index = 3%). Similar effects were seen in the other cells in our sample (e.g., Figure 2B, suppression index from 50% to 23%) and indeed in the average of all cells that showed some surround suppression (Figure 2C). On average, the suppression index decreased from 38% ± 3% (SEM, n = 60) when mice were stationary to 23% ± 3% during locomotion. Locomotion increased the responses more for large stimuli than for small stimuli, and this increase in response grew linearly with stimulus size, both for the example neurons (Figures 2D and 2E) and for the population (Figure 2F). Across cells, locomotion increased both the peak firing rate and the spontaneous firing rate (Figures 2G and 2H). Moreover, it markedly changed the properties of spatial integration, increasing the preferred stimulus size and decreasing the strength of size tuning (Figures 2I and 2J). These effects were consistent across cells, and they were similar in cells with broad or thin spikes (see Figure S1 available online), suggesting that they may affect all cells equally. Conversely, locomotion did not affect other properties of the responses, such as orientation tuning [9] (data not shown).

These effects of locomotion could not be explained by artifacts of eye movements (Figure S2). Eye movements were invariably horizontal and only slightly larger during locomotion (averaging 4.4° ± 3.9°, SD) than when the animal was stationary (2.5° ± 2.6°). These effects were too small to disrupt estimates of surround suppression: locomotion affects most the responses to the largest stimuli, which are one order of magnitude larger than typical eye movements. Moreover, eye movements were rare: the eye was within 5° of the central position in 66% of trials during locomotion and 78% of trials while the animals were stationary. Finally, the effects of locomotion were seen even in trials where the eye did not move. In these trials, locomotion reduced the suppression index from 38% ± 3% (SEM, n = 58) to 26% ± 3% (Figure S2). Similarly, the effects of locomotion were not due to artifactual changes in spike isolation (Figure S3) or to artifactual correlations between the visual preferences of the neurons (preferred orientation or receptive field center) and the properties of the stimulus (Figure S4).

**Figure 3:**
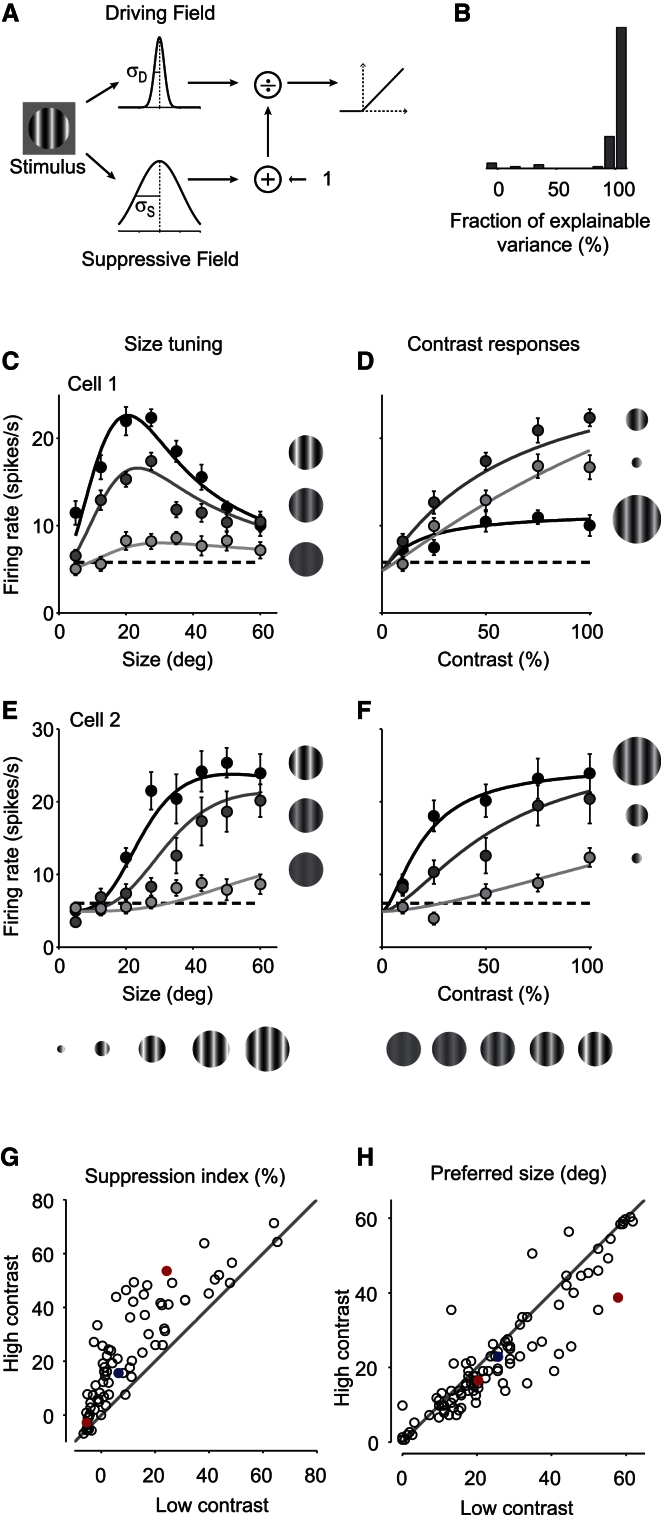
Divisive Normalization Describes Surround Suppression in Mouse V1 (A) Divisive model of spatial integration. (B) Percentage of the explainable variance captured by the model. First bin indicates values < 0, and the last bin indicates values > 100%. Ordinate/vertical axis is number of cells. (C) Responses of an example neuron to changes in stimulus size for three stimulus contrasts (light gray: 10%, darker gray: 50%, black: 100%). Curves are fits of the model. Error bars in (C)–(F) indicate SEM. (D) A different view of the same data, expressed as a function of stimulus contrast for three stimulus diameters (light gray: 13°, darker gray: 28°, black: 60°). Curves represent the fits of the same model as in (C). (E and F) Same as (C) and (D), for a different example neuron. Stimulus diameters in (F) are 20° (light gray), 35° (darker gray), and 60° (black). (G and H) Effects of contrast on spatial integration. As the stimulus contrast increases from 10% (abscissa) to 100% (ordinate/vertical axis), the suppression index increases (G) and the preferred stimulus size decreases (H). Red dots represent two example neurons presented in (C and D) and (E and F). Blue dots are the mean values for the whole population of cells.

Spatial integration by neurons in area V1 is known to depend strongly on contrast [11–13]. To fully describe how spatial integration is affected by locomotion, therefore, we sought to characterize it with stimuli varying in size and contrast. We considered a divisive normalization model (Figure 3A), where the responses are driven by the output of a “driving field” and suppressed by the output of a wider “suppressive field” [13, 14]. We modeled both fields as Gaussians and defined the model’s response to a stimulus with contrast *c* and diameter *d* as

$$R\left(c,d\right)=R_{0}+\frac{R_{D}C^{n}D{\left(d\right)}^{m}}{1+R_{s}c^{n}S{\left(d\right)}^{m}}.$$

Here *R*_0_ is the baseline (spontaneous) firing rate, *m* and *n* are exponents, *R*_*D*_ and *R*_*S*_ are the strengths of driving field and suppressive field, and *D*(*d*) and *S*(*d*) are the fractions of the driving field and suppressive field covered by the stimulus (between 0 and 1), which depend on the sizes σ_*D*_ and σ_*S*_ of the driving field and suppressive field. An additional parameter δ allows the model to account for slight miscentering of the stimuli. The model is therefore specified by eight parameters, and we obtained those parameters by fitting responses to 41 stimuli: all combinations of eight diameters and five contrasts, and a blank stimulus.

The model provided good fits to the responses, accounting for the effects of both size and contrast (Figures 3C–3F). With a single set of parameters, the model accounted for the increase in surround suppression with contrast (Figure 3C) and for the associated increase in contrast saturation with size (Figure 3D). Similar results were seen in the other cells (Figures 3E and 3F). For 99 of 105 neurons that we probed with combinations of stimulus size and contrast, the model accounted for >80% of the explainable variance (Figure 3B). We can therefore use the model to quantify the effects of contrast on spatial integration in awake mouse V1. By doing so, we observed two effects of increasing contrast: an increase in the strength of surround suppression (Figure 3G) and a decrease in the preferred stimulus size (Figure 3H). Similar effects have been observed in primates [11–13].

**Figure 4:**
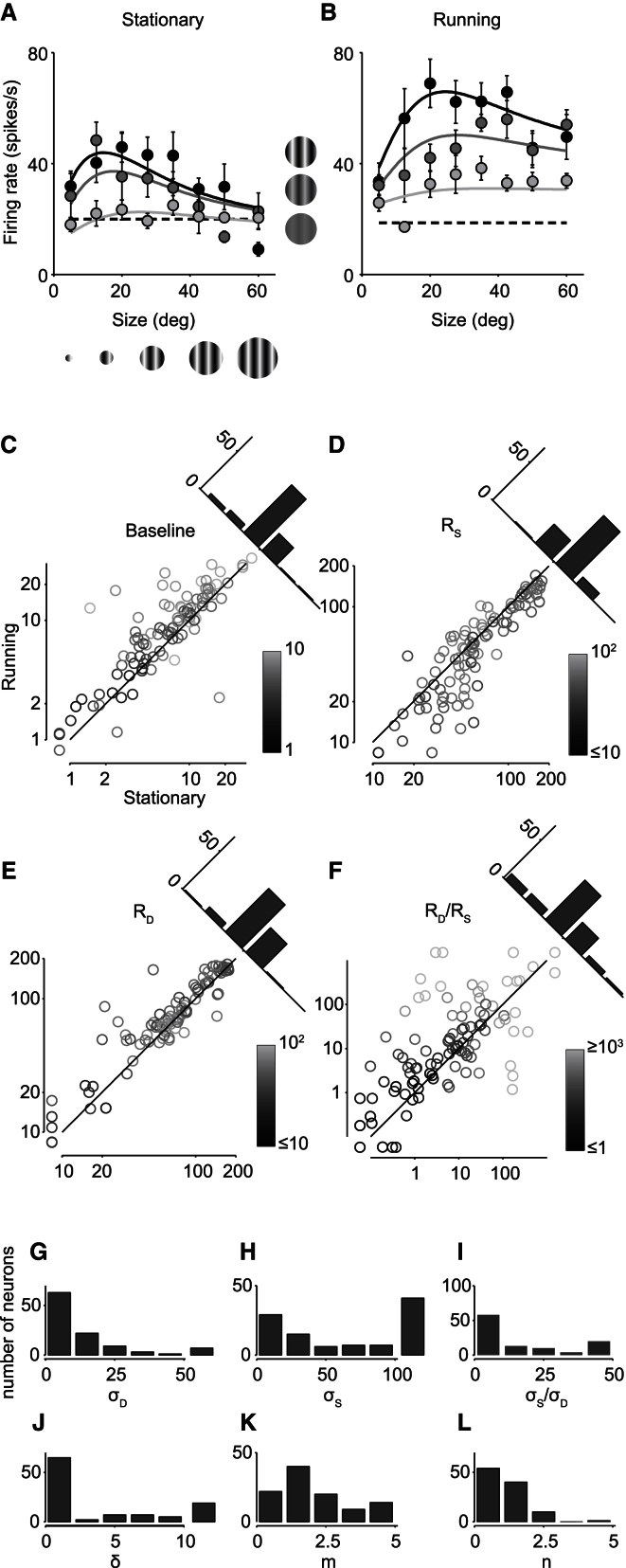
Effects of Locomotion on Divisive Normalization (A and B) Responses of an example neuron as a function of stimulus diameter (abscissa) and contrast (light gray: 10%, darker gray: 50%, black: 100%) while the mouse is stationary (A) and during locomotion (B). Error bars represent SEM. Curves are the fits of the divisive normalization model in which only three parameters are free to change with locomotion: the baseline firing rate *R*_0_, the strength of the driving field *R*_*D*_, and the strength of the suppressive field *R*_*S*_. For this example neuron, locomotion increased baseline activity *R*_0_ from 11 to 24 and decreased the strength of both driving field (*R*_*D*_ from 77 to 33) and suppressive field (*R*_*S*_ from 133 to 17). The remaining parameters were fixed (σ_*D*_ = 7.3, σ_*S*_ = 364, δ = 0.0, *m* = 1.2, n = 1.0 for this neuron). (C) Locomotion increased the baseline firing rate. Each point corresponds to a cell, and the gray level indicates confidence in the estimates. In (C)–(F), gray scale bars show log SD of each parameter estimate, and histograms indicate the distribution of log differences. (D–F) Similar to (C), effects of locomotion are shown on the strength of the suppressive field *R*_*S*_ (D), the strength of the driving field *R*_*D*_ (E), and the ratio between the two (F). (G–L) Distribution of fixed parameters across population, showing the extent of the driving Gaussian (σ_*D*_) (G), the suppressive Gaussian (σ_*S*_) (H), the ratio of these two (I), the miscentering parameter (δ) (J), and the exponents *m* and *n* (K) and (L).

We then applied the divisive normalization model to characterize the effects of locomotion (Figures 4A and 4B). We considered the 99 of 105 cells whose responses were well fitted by the normalization model (the model accounted for >80% of the explainable variance), and we fitted their responses separately depending on whether the animal was stationary or moving (Figures 4A and 4B). To minimize the number of free parameters, we allowed as few parameters as possible to vary with locomotion. We could achieve good fits as long as we allowed three parameters to vary: the baseline firing rate *R*_0_, the strength of the driving field *R*_*D*_, and the strength of the suppressive field *R*_*S*_. The remaining parameters (*m*, *n*, σ_*D*_, σ_*S*_, and δ) were kept constant across conditions (Figures 4G–4L).

Locomotion had two main effects: it increased the baseline firing rate, and it reduced the strength of the suppressive field relative to the driving field (Figures 4C–4F). Across cells, locomotion increased the baseline firing rate *R*_*0*_ by a factor of 1.3 ± 0.2 (median ± median absolute deviation, n = 99 neurons, Figure 4C), a significant increase (p < 0.005, sign test). Similarly, locomotion decreased the strength of the suppressive field *R*_*S*_, by a factor of 0.9 ± 0.1 (Figure 4D, p < 0.005). Locomotion also increased the strength of the driving field *R*_*D*_, by a factor of 1.1 ± 0.2 (p < 0.005). However, these changes in *R*_*D*_ traded inversely with changes in *R*_*S*_, leading to an apparent variability across cells. Indeed, locomotion increased the ratio *R*_*D*_/*R*_*S*_, by a median factor of 1.7 (Figure 4F, significantly >1, p < 0.005). At any given stimulus size, this ratio is a measure of responsiveness, and responsiveness was indeed increased by locomotion.

We have shown that locomotion modulates the responses of V1 neurons by changing their properties of spatial integration. In agreement with previous results [9], we found that locomotion increases both baseline activity and visually driven activity. By varying stimulus size, however, we discovered that this increase is largest for large stimuli and progressively smaller for smaller stimuli. Locomotion, in summary, profoundly reduces the strength of surround suppression experienced by V1 neurons.

These results indicate that locomotion has more than a simple modulatory effect. Previous data on the effects of locomotion were obtained at a fixed stimulus size and suggested that locomotion affects responses to all stimuli equally [9]. Such a modulatory role could in principle be given a number of simple explanations, including one as simple as an increase in brain temperature [19]. However, our finding that locomotion shapes spatial selectivity makes it unlikely to operate through a global quantity.

Our findings indicate that the effects of locomotion differ substantially from those of spatial attention. Spatial attention typically results in an increase in effective stimulus contrast and therefore in stimulus competition [4, 14]. In primate V1, increasing stimulus contrast increases the strength of surround suppression [11, 12]. Our measurements indicate that similar effects are present in mouse V1. However, these effects are opposite to those of locomotion, which reduces spatial competition. Therefore, locomotion does not seem to cause an increase in effective contrast. Its effects are likely to rely on mechanisms different from those affected by spatial attention.

Perhaps the effect of locomotion on mouse V1 does resemble the effect of spatial attention seen in primate V1, but only for attention delivered to the periphery of the visual field. Indeed, whereas near the fovea of primate V1 attention appears to strengthen surround suppression, in the periphery it appears to weaken it [5]. This similarity suggests that locomotion may operate on similar mechanisms as peripheral attention.

We found that in mouse V1, the spatial integration and its dependence of contrast could be well described by a simple divisive normalization model. This model had previously been used to summarize spatial integration and contrast integration in cat and primate [13, 14]. It is attractive because it can directly predict the interactions of stimulus contrast and size, without the need for additional parameters [14]. Our results suggest that mouse V1 performs similar computations and extend previous results obtained with stimuli of a single size [20].

The normalization model allowed us to summarize the effects of locomotion concisely: an increase in baseline activity and a decrease in the relative strength of the divisive suppressive field. This phenomenological description does not by itself indicate what mechanisms are at play, because the mechanisms that underlie normalization are currently unclear [14]. However, having a simple, closed-form equation that summarizes the effects of contrast, spatial extent, and locomotion can help guide the search for the underlying mechanisms.

One of the possible mechanisms at play is synaptic inhibition. There is debate as to whether inhibition mediates surround suppression [21, 22]. In mouse V1, however, there is convincing evidence that it does, at least for a class of inhibitory neurons that express somatostatin and are particularly active in the awake cortex [18]. Indeed, in awake mouse V1 there is strong synaptic inhibition from broad regions of visual field [23]. Perhaps these interneurons expressing somatostatin are less active during locomotion, thus explaining the decrease in surround suppression. Other classes of interneurons could also play a role. Niell and Stryker [9] found neurons with thin spikes that are suppressed by stimuli when the animal runs. We did not find such cells (in only 3 of 215 neurons were responses decreased by stimuli when the animal was running), but the effect is clear in their measurements and points to a class of interneurons expressing parvalbumin, because these neurons have thin spikes. Perhaps during locomotion, large stimuli suppress a larger number of these interneurons, thereby reducing the overall amount of inhibition. To elucidate these matters, future experiments will need to clarify whether and how each inhibitory cell class is affected by locomotion.

Conversely, our results seem to rule out the possible involvement of mechanisms relying on acetylcholine. In primate V1, acetylcholine does typically increase visual responses [24], but it also appears to increase the strength of surround suppression [25], which is the opposite of what we found for locomotion.

We do not know whether the effects of locomotion that we have uncovered in mouse also extend to other species such as cats, primates, or humans. If these effects are present also in the other species, then this points to an interaction of early vision and locomotion that has been hitherto unsuspected. If instead they are not, this may point to a fundamental difference between visual systems. Perhaps the visual system of the mouse is devoted to assisting the animal’s navigation more than to pattern vision and shape perception. In that case, it might be advantageous for the mouse visual cortex to have its spatial integration properties depend on locomotion, because this may assist in some spatiotemporal computations that are useful for navigation. However, the relevant computations need to be elucidated. The literature contains compelling theories for how surround suppression could help neural coding [26, 27], yet nothing in these theories seems to suggest that this suppression should be altered by locomotion.

## Experimental Procedures

All procedures were conducted in accordance with the UK Animals Scientific Procedures Act (1986). Experiments were performed at University College London under personal and project licenses released by the Home Office following appropriate ethics review. For detailed methods, please see the Supplemental Experimental Procedures.

## Supplemental Information

Document S1. Figures S1–S4 and Supplemental Experimental Procedures
