# Supplementary material for: Locomotion Controls Spatial Integration in Mouse Visual Cortex
Source: Curr Biol. 2013 May 20;23(10):890–4. doi: 10.1016/j.cub.2013.04.012 (PMC3661981; doi:10.1016/j.cub.2013.04.012)
Supplement: Document S1. Figures S1–S4 and Supplemental Experimental Procedures [file mmc1.pdf]

Current Biology, Volume *23*

Supplemental Information

Locomotion Controls Spatial Integration in Mouse Visual Cortex

Aslı Ayaz, Aman B. Saleem, Marieke L. Schölvinck, and Matteo Carandini

Supplemental Information

Supplemental Inventory

Supplemental Figures 2
Figure S1. Locomotion effects do not depend on spike shape. 2
Figure S2. Locomotion effects do not depend on eye movements. 3
Figure S3. Locomotion effects do not depend on quality of spike isolation. 4
Figure S4: Locomotion effects do not depend on stimulus position and orientation. 5
Supplemental Experimental Procedures 6
Physiology 6
Visual Stimulation 7
Locomotion 7
Eye Movements 7
Suppression Index 7
Divisive Model 8
Supplemental References 9

Supplemental Figures

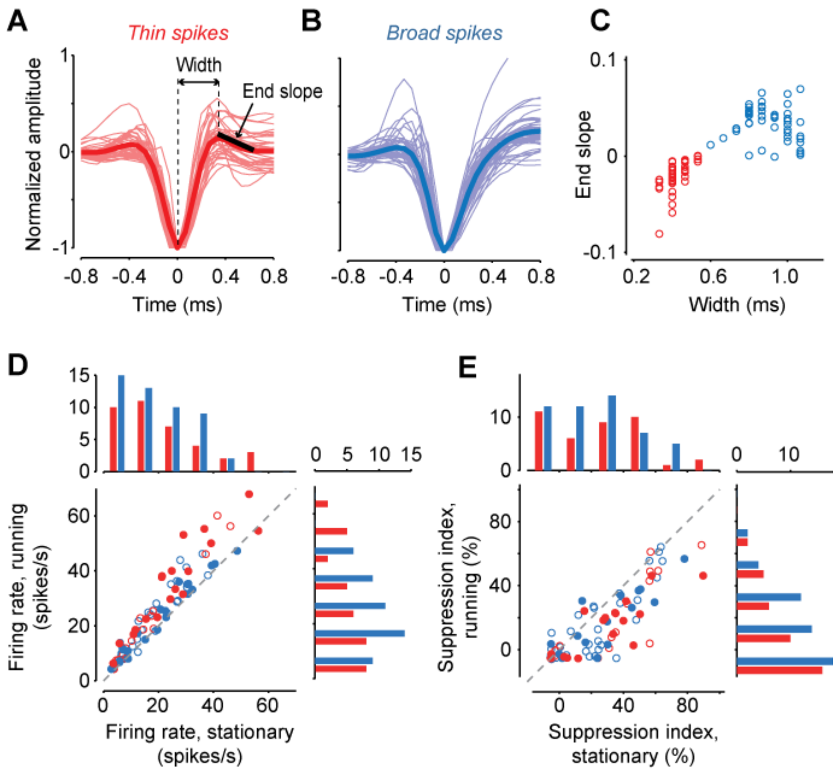

Figure S1. Locomotion Effects Do Not Depend on Spike Shape

To test whether the effects shown in Figure 2 differ among neurons of different types, we classified neurons based on the shape of their spike. Spike waveforms were normalized by their negative peak value and overlaid at negative peak (designated as 0 time point). Spike shapes were clustered into thin (**A**) and broad (**B**) spikes according to the width and end slope of their spike. Spike width is defined as the time difference between the trough of the spike shape and the peak after the trough. The end slope is the slope of the normalized spike trace at 0.47 ms after the negative peak. For all panels *red* and *blue* represent thin and broad spikes.

(**A**) The spike traces of cells with thin spikes (*light red*) and their mean (*dark red*).

(**B**) Same , for cells with broad spikes.

(**C**) Spike shape clustering of 89 neurons shown in Figure 2.

(**D**) Comparison of the effects of locomotion on firing rates at preferred stimulus size for thin and broad spiking neurons. *Solid dots* are well isolated units (see figure S3). Histograms show distributions for each cluster at stationary and locomotion states.

(**E**) Effects of locomotion on suppression indices.

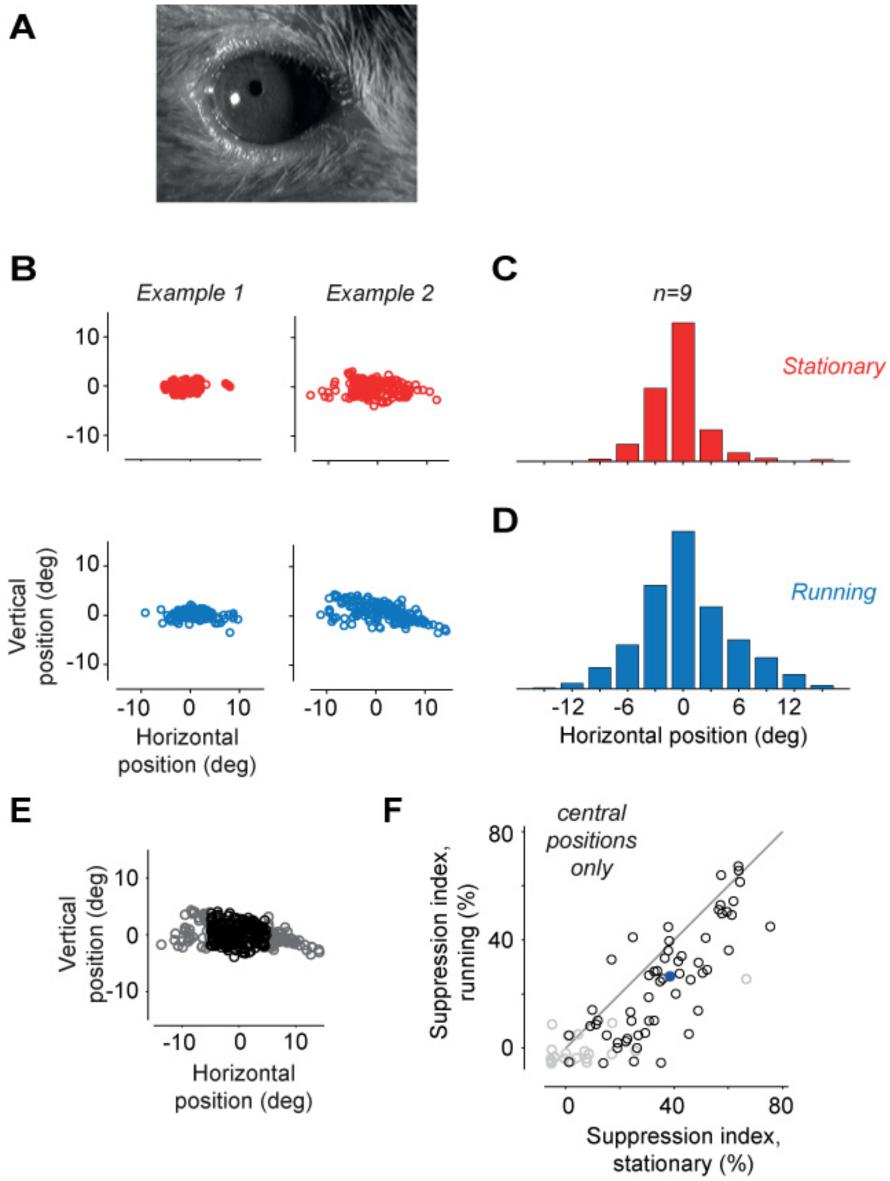

Figure S2. Locomotion Effects Do Not Depend on Eye Movements

To test whether the effects shown in Figure 2 may be influenced by eye movements, we measured eye position and performed a number of analyses.

(**A**) Eye position was measured by tracking the pupil using an infrared camera and analyzed with in-house Matlab software.

(**B**) Pupil position for two example sessions. X axis is the horizontal position and y axis is the vertical position.

(**C**) Distribution of pupil positions in the horizontal dimension during stationary trials for 9 recording sessions for 4 mice.

(**D**) Same as **C** but during locomotion.

(**E**) Pupil positions of an example session with larger eye movements (example 2 in B). Black dots are the selected central pupil positions (< 5 degree from the center of all pupil positions) and the rest are in gray.

(**F**) Comparison of suppression indices during locomotion and stationary trials, similar to Figure 2H, only considering trials with central pupil positions (< 5 degrees from the center on the horizontal dimension).

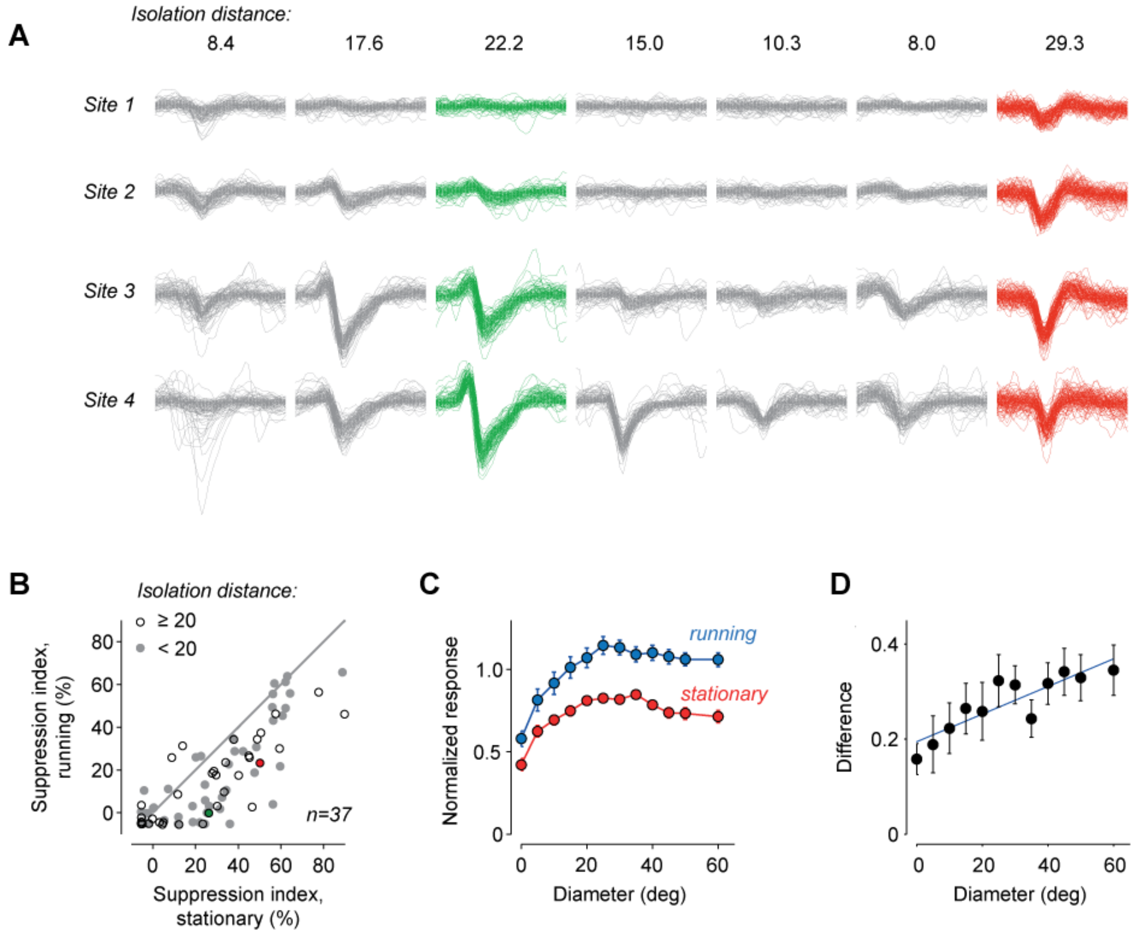

Figure S3. Locomotion Effects Do Not Depend on Quality of Spike Isolation

To test whether the effects shown in Figure 2 apply also to single neurons, we performed careful spike sorting, and repeated the analysis focusing on neurons with well-isolated spikes.

(**A**) Sample traces recorded on 4 sites of the linear probe which are 50 µm apart. The bottom row lists the isolation distances of each unit. These distances are calculated by the KlustaKwik algorithm. We chose a very conservative threshold of 20 to indicate well-isolated units (*green* and *red* units). The unit with red spikes is the one in Figure 2**B**.

(**B**) Effect of locomotion on suppression indices for well isolated units (*black circles* n=37/89) and the rest (*gray circles*). *Red* and *green dots* are sample units in **A**. The key finding that surround suppression decreases with locomotion holds for both well- isolated and poorly-isolated units.

(**C**) Comparison of size tuning curves during locomotion (*blue*) and stationary (*red*) trials, averaged over the units with best isolation (marked in *black* in **B**). Error bars in (C) and (D) represent SEM.

(**D**) Difference in responses between the two conditions, for the data in **C**.

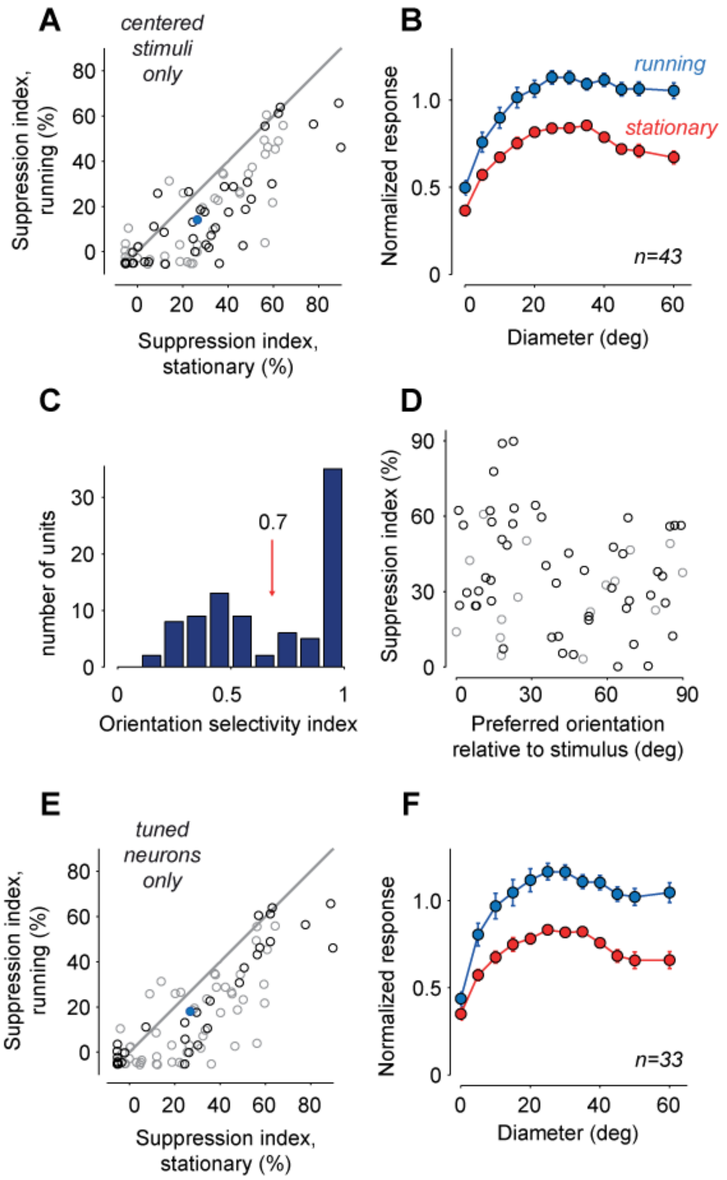

Figure S4: Locomotion Effects Do Not Depend on Stimulus Position and Orientation

To test whether the effects shown in Figure 2 depend on the precise attributes of the visual stimuli, we analyzed responses separately depending on stimulus orientation and position.

(**A**) Effects of locomotion on suppression indices. *Black dots* represent units with receptive field centers within 5 degrees of the stimulus center (n=43/89), the rest are in *gray*. *Blue dot* shows the mean value for black dots.

(**B**) Population average of size tuning curves during locomotion (*blue*) and stationary (*red*) trials for precisely centered stimuli (for units in *black* in **A**). Error bars in (B) and (F) represent SEM.

(**C**) Orientation selectivity indices (OSI) of all units. OSI distribution is as expected [1], with mean equal to 0.7.

(**D**) Suppression indices during stationary trials vs. the difference between the stimulus orientation and the preferred orientation of the neurons. *Black dots* are units that are tuned for orientation (OSI > 0.4) rest are in *gray*. (p=0.47 for orientation selective units)

(**E**) Effect of locomotion on suppression indices for units with OSI > 0.4 and the difference between their preferred orientation and stimulus orientation is <30 degrees (black dots n=33). These units have similar locomotion effects compared to rest of the population.

(**F**) Size tuning curves during locomotion and stationary trials for selected units (*black dots* in **E**).

Supplemental Experimental Procedures

Physiology

13 adult wild-type mice (C57BL/6J, 20–35 g) were implanted with a custom-designed headpost and recording chamber over the left visual cortex using dental acrylic (Superbond C&B; Prestige Dental) under isoflurane anesthesia (4-5% induction, 1–2% maintenance). A ~1 mm^2 craniotomy was performed centered at coordinates 2.5 - 3 mm lateral from the midline and 0.5 mm anterior from lambda. The chamber was sealed with Kwik-Cast (WPI). The animal was administered an analgesic (Rimadyl, 1 µl/g s.c.) during surgery and for 3 days after surgery (Rimadyl, oral route). Following recovery (1 week), the animals were handled and acclimatized to the recording conditions. Mice were head-fixed and placed on an air-suspended Styrofoam ball [1-3]. Extracellular neural activity was recorded by advancing a 16-channel silicon probe into visual cortex (model A1X16-3mm-50-413; NeuroNexus Tech) to a depth of 900 um. The tissue was allowed to settle for 20 min before the start of each 2–3 h recording session. After each session, the recording chamber was resealed and the animal returned to the home cage until the subsequent recording session (mean 2, maximum 4 sessions per animal).

We collected two data sets: size tuning and size-contrast data sets with two different stimuli conditions (see section below for details of stimuli). In size tuning data set all units were obtained using autoclustering algorithm KlustaKwik followed by manual pruning. Recordings were filtered (0.3 - 5 kHz), threshold crossings were auto clustered using KlustaKwik [4] followed by manual adjustment using Klusters [5]. For a subset of size-contrast experiments, 56/105 units, were selected using manually adjusted threshold crossings and custom clustering software. We measured spike isolation quality [6], and based our analysis both the activity of well-isolated single neurons and multi-unit activity, as this did not change the result (Figure S3). In our recordings, units were predominantly localized to layer IV and layer V.

In our analysis we considered units that satisfied following criteria: a) mean response larger than 1 spikes/s; b) maximum response> 3 spikes/s; c) signal to noise ratio (mean/std of the mean) larger than 1.5; and d) receptive field center within 15˚ of the center of presented gratings. For size tuning data set 89/215 units and in size-contrast data set 105/330 units satisfied these criteria.

Specifically, Figure 1 presents results from the neurons where we measured size tuning (89 units) and groups them into tuned (60/89) and untuned (29/89). In Figure 2B, the population average focuses on the tuned neurons (60/89). In Figures 3 and 4 we report on a larger data set of neurons where we varied both size and contrast (105 neurons). Figure 4 shows 99/105 neurons for which the model explained more than 80 % of explainable variance. Eye movements were analyzed for all but one size tuning experiment (we failed to acquire eye movement data for one session). Thus the scatter plot in Figure S2**F** considers 83 units.

To compute the Orientation Selectivity Index, we fit the responses with the sum of two Gaussians whose centers are 180 degrees apart. Using this fit, the OSI is defined as:

$$OSI = \frac{R_{pref} - R_{orth}}{R_{pref} + R_{orth}}$$

where R_{pref} is response to the preferred orientation and R_{orth} is response to the orthogonal orientation (both are baseline subtracted).

Visual Stimulation

Visual stimuli were presented using custom-written software across three calibrated LCD monitors (HA191, Hanns.G, mean luminance 50 cd/m^2), covering an angle of 180° horizontal (divided in 3 monitors covering 60 deg each) and 45° vertical, 35 cm away from the eye. As a compromise for the differences of horizontal and vertical stimulus range, we chose stimuli that had at most 60 deg diameter. Initially we mapped receptive field center using flashing black and white bars across visual space. After determining the receptive field center we recorded spike responses to drifting sinusoidal gratings of 12 different orientations with a spatial frequency of 0.05 cycles/deg and a temporal frequency of 2 Hz, presented for 1 s over the receptive field. Inter-stimulus intervals with mid gray screen were 0.5 s long. Receptive field center and orientation of the stimuli were optimized for the multiunit activity recorded in one electrode site and used for the subsequent spatial integration stimuli (spatial and temporal frequency were kept the same). We used two sets of stimuli to study spatial integration in awake mice. In one data set (size-contrast) we presented gratings of 5 different contrasts and 8 stimulus diameters (10-16 repeats). In the other data set (size tuning) we presented sinusoidal gratings of 11 different diameters ranging between 5 degrees to 60 degrees of high contrast (40 repeats).

Locomotion

Movement of the ball was tracked with two optical mice. The average movement for each trial was calculated and used to classify trials as running or a stationary trial. The threshold for locomotion was set to 1.2 cm/s, a value that separated two modes in the distribution of speeds.

Eye Movements

We imaged eye movements with an infrared camera (DMK 21BU04.H, Imaging Source) with zoom lens (MVL7000, Navitar), positioned above the monitors. We analyzed images of the eyes with custom software that fitted the pupil with an ellipse and tracked its center. For each stimulus presentation (1 s) we computed a single pupil center, which is the mean of pupil centers in acquired images. This is a reasonable approximation since pupil movements are rare.

We then calculated the angular eye movements by

$$\theta_h = arcsin(dx/r)$$
$$\theta_v = arcsin(dy/r)$$

where θ_h, θ_v are horizontal and vertical angular eye movements and dx,dy are displacement of the pupil center from the center of all pupil positions in the image plane within a session. Finally r is the radius of the mouse eye (1.6 mm, Ref. [7]).

Suppression Index

We measured the degree of suppression due to increases in stimulus diameter with a suppression index (SI):

$$SI = \frac{(R_P - R_L)}{R_P}$$

Here R_P and R_L are the responses of the neuron at the preferred stimulus size and at the largest stimulus size. R_P and R_L were computed using the model fits to the diameter responses. We define the preferred size R_P as the smallest stimulus size at which neuron's response equals to 95 % of the maximal response. Please note that we choose the preferred size at 95 % of the maximal response instead of the absolute maximal response because this allows us to investigate whether the saturation point for untuned cells change under different conditions.

Divisive Model

Responses of neurons are fit with a divisive normalization model that is a modified version of "Ratio of Gaussians" model that has been proposed to explain surround suppression [8]. Models were fit by minimizing mean squared error. In the model, two Gaussian fields (for simplicity, one-dimensional) provide input to each neuron: a driving field G_D and a suppressive field G_S. Their responses to stimuli with diameter d are

$$D(d) = \int_{-d+\delta}^{d+\delta} G_D(x)dx = \operatorname{erf}\left(\frac{d+\delta}{\sqrt{2}\sigma_D}\right) + \operatorname{erf}\left(\frac{d-\delta}{\sqrt{2}\sigma_D}\right)$$

$$S(d) = \int_{-d+\delta}^{d+\delta} G_S(x)dx = \operatorname{erf}\left(\frac{d+\delta}{\sqrt{2}\sigma_S}\right) + \operatorname{erf}\left(\frac{d-\delta}{\sqrt{2}\sigma_S}\right)$$

where σ_D and σ_S are the extents of the two fields, and δ is a parameter that accounts for slight miscentering of the stimulus relative to the center of the fields.

A neuron's response is computed as the ratio of the field responss:

$$R(c,d) = R_0 + \frac{R_D c^n D(d)^m}{1 + R_S c^n S(d)^m}$$

Here R_0 is the baseline (spontaneous) firing rate, m and n are exponents, R_D and R_S are the strengths of driving field and suppressive field.

We estimated the quality of the model predictions by calculating the percent of explainable (stimulus) variance accounted for by the model [9].

$$\beta = \frac{\sigma_r^2 - \sigma_e^2}{\sigma_r^2 - \sigma_\eta^2}$$

Where σ_r^2 is the response power assuming zero mean, σ_e^2 is the mean squared error between the model estimated and measured response, and σ_η^2 is the estimated variance due to the noise in the data.

$$\sigma_r^2 = \langle \frac{1}{M} \sum_i r_i^2 \rangle$$

$$\sigma_e^2 = \langle \frac{1}{M} \sum_i (r_i - \hat{r}_i)^2 \rangle$$

$$\sigma_\eta^2 = \frac{n}{n-1}\left[\langle \frac{1}{M} \sum_i {r_i}^2 \rangle - \frac{1}{M} \sum_i \langle r_i \rangle^2\right]$$

In these equations M is the number of stimulus conditions, square brackets are trial averages, and n is the number of trials for each stimulus.

Unlike the percentage of explained variance, the percentage of explainable variance can occasionally go above 100%. This simply occurs when the noisiness of the data has been overestimated. In addition, the percentage of the explainable variance can go below zero, for models that are worse than a straight line going through the mean of the data.

In Figure 4 we fit data with a model in which only three parameters were free to change with locomotion: the baseline firing rate R_0, the strength of the driving field R_D, and the strength of the suppressive field R_S.The remaining parameters (m, n, σ_D and σ_S) were kept constant and their values were obtained from fits to the whole data set including both locomotion and stationary trials. To compare the effects of locomotion on three free parameters we fit the reduced model independently for locomotion and stationary trials of 100 bootstrap samples of the data. From these estimates we computed the confidence on each parameter as the mean standard deviation, δ:

$$\delta = \sqrt{\left({\delta_R}^2 + {\delta_S}^2\right)/_2}$$

Where δ_R and δ_S are standard deviation of parameters obtained from fits to locomotion and stationary trials. This mean deviation is used to color code confidence on parameter estimates in Figure 4.

Supplemental References

1. Niell, C.M., and Stryker, M.P. (2010). Modulation of visual responses by behavioral state in mouse visual cortex. Neuron *65*, 472-479.
2. Harvey, C.D., Collman, F., Dombeck, D.A., and Tank, D.W. (2009). Intracellular dynamics of hippocampal place cells during virtual navigation. Nature *461*, 941-946.
3. Dombeck, D.A., Khabbaz, A.N., Collman, F., Adelman, T.L., and Tank, D.W. (2007). Imaging large-scale neural activity with cellular resolution in awake, mobile mice. Neuron *56*, 43-57.
4. Harris, K.D., Henze, D.A., Csicsvari, J., Hirase, H., and Buzsaki, G. (2000). Accuracy of tetrode spike separation as determined by simultaneous intracellular and extracellular measurements. J Neurophysiol *84*, 401-414.
5. Hazan, L., Zugaro, M., and Buzsaki, G. (2006). Klusters, NeuroScope, NDManager: a free software suite for neurophysiological data processing and visualization. J Neurosci Methods *155*, 207-216.
6. Schmitzer-Torbert, N., Jackson, J., Henze, D., Harris, K., and Redish, A.D. (2005). Quantitative measures of cluster quality for use in extracellular recordings. Neuroscience *131*, 1-11.
7. Remtulla, S., and Hallett, P.E. (1985). A schematic eye for the mouse, and comparisons with the rat. Vision Res *25*, 21-31.
8. Cavanaugh, J.R., Bair, W., and Movshon, J.A. (2002). Nature and interaction of signals from the receptive field center and surround in macaque V1 neurons. J Neurophysiol *88*, 2530-2546.
9. Sahani, M., and Linden, J.F. (2003). How linear are auditory cortical responses? In Advances in neural information processing systems, Volume 15, S. Becker, S. Thrun and K. Obermayer, eds. (Cambridge, MA: MIT Press), pp. 125-132.
